# *Staphylococcus aureus *Biofilm and Planktonic cultures differentially impact gene expression, mapk phosphorylation, and cytokine production in human keratinocytes

**DOI:** 10.1186/1471-2180-11-143

**Published:** 2011-06-21

**Authors:** Patrick R Secor, Garth A James, Philip Fleckman, John E Olerud, Kate McInnerney, Philip S Stewart

**Affiliations:** 1 Center for Biofilm Engineering, Montana State University, Bozeman, Montana, USA; 2 Division of Dermatology, University of Washington, Seattle, Washington, USA; 3 Functional Genomics Core Facility, Montana State University, Bozeman, Montana, USA

## Abstract

**Background:**

Many chronic diseases, such as non-healing wounds are characterized by prolonged inflammation and respond poorly to conventional treatment. Bacterial biofilms are a major impediment to wound healing. Persistent infection of the skin allows the formation of complex bacterial communities termed biofilm. Bacteria living in biofilms are phenotypically distinct from their planktonic counterparts and are orders of magnitude more resistant to antibiotics, host immune response, and environmental stress. *Staphylococcus aureus *is prevalent in cutaneous infections such as chronic wounds and is an important human pathogen.

**Results:**

The impact of *S. aureus *soluble products in biofilm-conditioned medium (BCM) or in planktonic-conditioned medium (PCM) on human keratinocytes was investigated. Proteomic analysis of BCM and PCM revealed differential protein compositions with PCM containing several enzymes involved in glycolysis. Global gene expression of keratinocytes exposed to biofilm and planktonic *S. aureus *was analyzed after four hours of exposure. Gene ontology terms associated with responses to bacteria, inflammation, apoptosis, chemotaxis, and signal transduction were enriched in BCM treated keratinocytes. Several transcripts encoding cytokines were also upregulated by BCM after four hours. ELISA analysis of cytokines confirmed microarray results at four hours and revealed that after 24 hours of exposure, *S. aureus *biofilm induced sustained low level cytokine production compared to near exponential increases of cytokines in planktonic treated keratinocytes. The reduction in cytokines produced by keratinocytes exposed to biofilm was accompanied by suppressed phosphorylation of MAPKs. Chemical inhibition of MAPKs did not drastically reduce cytokine production in BCM-treated keratinocytes suggesting that the majority of cytokine production is mediated through MAPK-independent mechanisms.

**Conclusions:**

Collectively the results indicate that *S. aureus *biofilms induce a distinct inflammatory response compared to their planktonic counterparts. The differential gene expression and production of inflammatory cytokines by biofilm and planktonic cultures in keratinocytes could have implications for the formation and persistence of chronic wounds. The formation of a biofilm should be considered in any study investigating host response to bacteria.

## Background

In many environments bacteria exist as a complex, multi-species surface-associated community termed biofilm. Bacteria within these communities secrete an extracellular polymer matrix, form complex structures, and are phenotypically distinct from their planktonic counterparts [1,2], and are orders of magnitude more resistant to antibiotics and biocides than planktonic bacteria [3]. Furthermore, bacterial genes involved in biofilm formation are controlled by regulatory systems that also control the expression of virulence factors [4,5].

Bacterial biofilms are a major barrier to healing in chronic wounds. In patients with underlying disease (i.e. diabetes, pulmonary disease), wounded epithelium offers an ideal environment for bacteria to form a biofilm due to susceptibility to contamination, availability of nutrients, and abundant surface area for attachment. Chronic-wound biofilms are not cleared by the host's immune system and are resistant to traditional treatment strategies such as antibiotics [6]. Cutaneous wounds progress through three highly regulated phases of wound repair: inflammation, epithelialization, and tissue remodeling. Chronic wounds display abnormal progression through these phases including prolonged inflammation and failure to re-epithelialize. Currently, removal of the biofilm by frequent debridement is one of the most clinically effective treatments applied to chronic wounds [7]. A recent study showed that biofilms were prevalent in chronic wounds and rare in acute wounds [8], but the role biofilms play in preventing wound healing and mechanisms involved have yet to be determined.

*S. aureus *is an important human pathogen associated with numerous skin diseases including chronic-wound infections. *S. aureus *produces a wide range of virulence factors including hemotoxins, pore forming toxins, and superantigens (e.g. toxic shock syndrome toxin-1, *Staphylococcal *enterotoxin). The impact of biofilm formation on *S. aureus *virulence is controversial. In one study, virulence factor gene expression in *S. aureus *cells within a biofilm was shown to be downregulated when compared to planktonic *S. aureus *cultures [2]. Another study showed that biofilm formation had no effect on the virulence of *S. aureus *[9], while several studies highlight the necessity of regulatory elements associated with biofilm formation on the regulation of virulence [10,11].

Human keratinocytes (HKs) are the most abundant cell type in the epidermis and are essential for wound healing. HKs are constantly exposed to bacterial stimuli and function in innate immunity through the formation of a physical barrier to the external environment and the recognition of conserved pathogen associated molecular patterns (PAMPs). Examples of PAMPs include the bacterial cell wall components peptidoglycan and lipoteichoic acid, bacterial DNA, flagella, and other conserved structures [12]. PAMPs are recognized by cell surface receptors called toll like receptors (TLRs) which are found on a variety of cell types including professional immune cells, endothelial cells, and cells of the epidermis. HKs express functional TLRs making them the first line of defense against bacteria in the skin [13]. HK activation induced by TLRs in response to bacterial stimuli is mediated in part by mitogen activated protein kinase (MAPK; specifically JNK, p38, and ERK) cascades resulting in the production of inflammatory cytokines [14-16]. MAPKs are major components regulating the pathology of chronic inflammation, diabetes mellitus, and other chronic diseases [17,18]. The highly orchestrated production of inflammatory cytokines by HKs is an important initial step in a normal immune response. Derangement of cytokine production by bacterial infection can lead to chronic inflammatory conditions [19].

In this study, we investigated the transcriptional response of HKs exposed to *S. aureus *biofilm conditioned medium (BCM) and planktonic conditioned medium (PCM) to reveal genes associated with pathogenesis. We correlated microarray data with data from enzyme-linked immunoassays (ELISA) and enzyme inhibition assays, to delineate a biofilm specific response associated with inflammation in HKs and formulate a hypothesis for biofilm-induced pathogenesis in chronic wounds.

## Results

### Proteomic analysis of BCM and PCM

**Figure 1:**
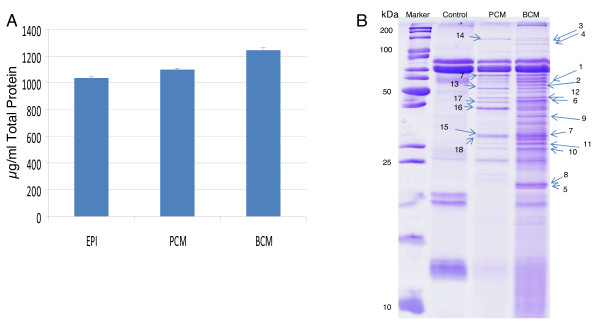
**1D SDS - PAGE and Total Protein Concentration in BCM and PCM**. The total protein concentration in BCM and PCM did not differ drastically (A), but several differences in the extracellular proteome of planktonic and biofilm cultures of *S. aureus *were revealed by 1D SDS-PAGE (B). The presence of a smear and low molecular weight peptides in the BCM indicates the presence of a bacterial protease. Bands in (B) marked with an arrow were excised and analyzed by HPLC-MS/MS (Table 1).

A preliminary proteomic analysis of BCM and PCM revealed differential protein compositions. BCM contained slightly more total protein than PCM and analysis by 1D gel electrophoresis revealed BCM contained a more complex banding pattern than PCM (Figure 1). Additionally, smearing was consistently observed in the BCM possibly indicating the presence of a bacterial protease. Protein identification of selected bands by mass spectrometry is listed in Table 1. PCM was found to contain several enzymes involved in glycolysis while BCM contained proteins relating to translation in addition to proteins which were not identified by a Mascot search.

**Table 1:** Proteins identified by HPLC-MS/MS

Band #	Sample	NCBI Accession	Name	Function
1	BCM	gi15924466	30S ribosomal protein S1 [*Staphylococcus aureus *subsp. aureus Mu50]	translation
1	BCM	gi227557405	elongation factor G [*Staphylococcus aureus *subsp. *aureus *MN8]	translation
2	BCM	gi15923949	glycerophosphoryl diester hosphodiesterase [*Staphylococcus aureus *subsp. *aureus *Mu50]	glycerophospholipid metabolism
3	BCM	gi15924653	valyl-tRNA synthetase [*Staphylococcus aureus *subsp. *aureus *Mu50]	translation
4	BCM	gi258423763	isoleucyl-tRNA synthetase Staphylococcus aureus A9635]	translation
5	BCM	gi2506027	N-acetyl-glucosaminidase [*Staphylococcus aureus*]	exoglycosidase
6	BCM	gi15924060	amidophosphoribosyltransferase *Staphylococcus aureus *subsp. *aureus *Mu50]	purine nucleotide biosynthesis
7	BCM	gi128852	Staphylococcal nuclease	nuclease
8	BCM	No significant hits	NA	NA
9	BCM	gi258424814	catalase [*Staphylococcus aureus *A9635]	antioxidant/oxidative stress
9	BCM	gi21282950	catalase [*Staphylococcus aureus *subsp. *aureus *MW2]	antioxidant/oxidative stress
10	BCM	No significant hits	NA	NA
11	BCM	No significant hits	NA	NA
12	BCM&PCM	gi15925406	phosphoglycerate mutase [*Staphylococcus aureus *subsp. *aureus *Mu50]	glycolysis
12	BCM&PCM	gi282917765	2,3-bisphosphoglycerate-dependent phosphoglycerate mutase [*Staphylococcus aureus *subsp. *aureus *D139]	glycolysis
12	BCM&PCM	gi|15927092	6-phosphogluconate dehydrogenase [*Staphylococcus aureus *subsp. *aureus *N315]	Pentose phosphate
			bifunctional 3-deoxy-7-hosphoheptulonate	
12	BCM&PCM	gi15924727	synthase/chorismate mutase [*Staphylococcus aureus *subsp.	shikimate pathway
			*aureus *Mu50]	
12	BCM&PCM	gi15923310	glycerol ester hydrolase [*Staphylococcus aureus *subsp. *aureus *Mu50]	lipase
13	BCM&PCM	gi15924543	superoxide dismutase [*Staphylococcus aureus *subsp. *aureus *Mu50]	antioxidant/oxidative stress
14	BCM&PCM	gi15923346	5-methyltetrahydropteroyltriglutamate--homocysteine S-methyltransferase [*Staphylococcus aureus *subsp. *aureus *Mu50]	methionine metabolism
14	BCM&PCM	gi293501167	aconitate hydratase 1 [*Staphylococcus aureus *subsp. *aureus *58-424]	TCA
15	PCM	gi15925596	fructose-1,6-bisphosphate aldolase [*Staphylococcus aureus *subsp. *aureus *Mu50]	glycolysis
16	PCM	gi15923621	lipoprotein [*Staphylococcus aureus *subsp. *aureus *Mu50]	cell wall component
16	PCM	gi15925115	fructose-bisphosphate aldolase [*Staphylococcus aureus *subsp. *aureus *Mu50]	glycolysis
17	PCM	gi289550260	fructose-bisphosphate aldolase class II [*Staphylococcus lugdunensis *HKU09-01]	glycolysis
17	PCM	gi283470068	phosphoglycerate kinase [*Staphylococcus aureus *subsp. *aureus *ST398]	glycolysis
18	PCM	gi15923952	glucose-6-phosphate isomerase [*Staphylococcus aureus *subsp. *aureus *Mu50]	glycolysis
18	PCM	gi15923762	glyceraldehyde-3-phosphate dehydrogenase [*Staphylococcus aureus *subsp. *aureus *Mu50]	glycolysis
18	PCM	gi151221290	ornithine carbamoyltransferase [*Staphylococcus aureus *subsp. *aureus *str. Newman]	urea cycle

Proteins identified by HPLC-MS/MS analysis. Band numbers represent excised bands from 1D-SDS PAGE analysis of BCM and PCM (Figure 1).

### *S. aureus *BCM upregulates genes associated with inflammation and apoptosis in human keratinocytes

The transcriptional response of HKs exposed to *S. aureus *PCM and BCM were examined. HKs were exposed to BCM and PCM for four hours prior to microarray analysis. Our previous results indicated that after four hours of exposure to BCM, HKs undergo cytoskeletal rearrangements including the formation of filopodial structures and rounding of the cell body, but have not started late-stage apoptotic programs [20].

Transcriptional analysis revealed that BCM upregulated 65 transcripts and downregulated 247 transcripts at least 1.5 fold (p < 0.01) compared to PCM (Additional file 1). Some of the most highly upregulated transcripts by BCM included (i) activated protein-1 (AP-1) family members (*fos*, *atf*, *jun*), (ii) *egr1 *stress response transcription factor, and (iii) cytokines. The calcium-binding protein S100P, which has been described as diagnostic for chronic inflammation [21], was also found to be upregulated 2.2 fold by BCM compared to PCM. Nuclear factor kappa B (NFkB) negative regulators TNFAIP3 (A20) and NFkBIA were also upregulated in BCM-treated HKs, indicating active regulation of this important inflammatory pathway.

**Figure 2:**
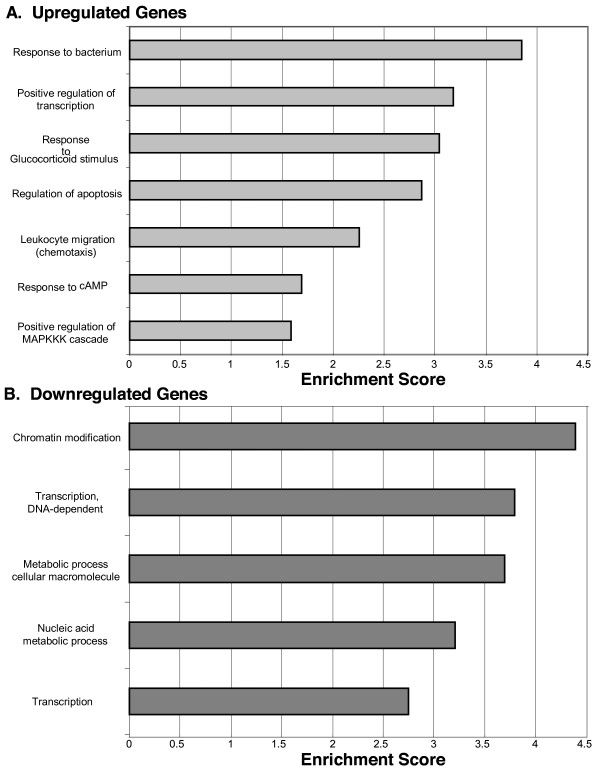
**Functional clustering of BCM induced genes**. Functional terms significantly associated (p < 0.05, Benjamini correction for multiple testing) with BCM induced genes relative to PCM induced genes. Functional annotation clusters with an enrichment score greater than 1.5 were considered significant. (A) Analysis of significantly upregulated genes (fold change ≥1.5) revealed functional annotation clusters associated with response to bacteria and external stimuli, apoptosis, immune response and inflammation, and signal transduction. (B) Analysis of significantly downregulated genes (fold change ≤1.5) revealed functional annotation clusters associated with chromatin modification, transcription, and metabolism.

An enrichment analysis was conducted using The Database for Annotation, Visualization and Integrated Discovery (DAVID) functional annotation clustering tool to identify over-represented (p < 0.05; Benjamini Hochberg correction for multiple testing) gene ontology terms. Seven functional annotation clusters with enrichment scores greater than 1.5 were identified in upregulated transcripts while five functional annotation clusters were identified in downregulated genes. Over-represented clusters in the upregulated transcript list contained terms relating to response to bacteria and external stimuli, apoptosis, immune response and inflammation, and signal transduction (Figure 2). Over-represented clusters in the downregulated transcript list contained terms associated with chromatin modification, transcription, and metabolism.

### *S. aureus *BCM induces apoptosis in HKs

**Figure 3:**
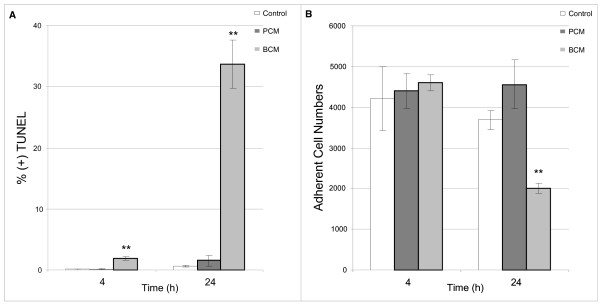
**BCM induces apoptosis and cell detachment in HKs**. (A) Percentage of HKs staining positive for TUNEL. BCM induces significant levels of apoptosis in HKs after 4 and 24 hours of exposure while PCM does not. TUNEL data represents positive TUNEL cell counts over total cell counts. (B) Total cell counts obtained from propidium iodide stained HKs. After 24 hours of exposure to BCM, roughly half of the BCM treated HKs were still adhering to the culture well. Results represented as mean ± SD, n = 4, ** p < 0.01.

Enrichment analysis of microarray data indicated genes relating to apoptosis were over-represented in BCM treated HKs. Apoptosis was confirmed using a TUNEL assay. A significant percentage of BCM treated HKs were undergoing apoptosis at four and 24 hours while the percentage of apoptotic PCM treated HKs was not significantly different from control cells (Figure 3A). Additionally, a significant decrease in adherent cell numbers was observed after 24 hours of exposure to BCM which was not observed in PCM treated HKs (Figure 3B).

### *S. aureus *PCM induces higher levels of cytokine production relative to BCM in human keratinocytes

**Figure 4:**
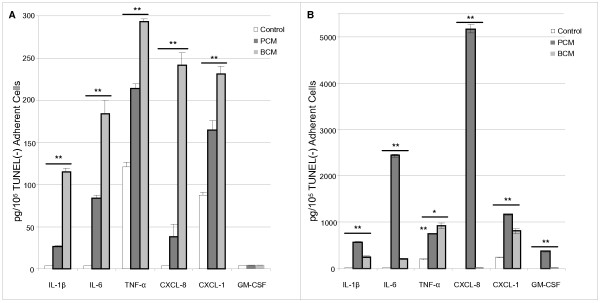
**Cytokine production in adherent, non - apoptotic HKs exposed to BCM or PCM**. BCM induces more cytokines per adherent, non-apoptotic cell after four hours while PCM induces more cytokines per adherent, non-apoptotic cell after 24 hours. Cytokine levels in HKs after 4 (A) and 24 hours (B) of exposure to PCM, BCM, or Control. Data normalized to pg protein/100,000, TUNEL negative, adherent cells. Results represented as mean ± SD, n = 3, *p < 0.05, **p < 0.01.

Several of the most significantly upregulated genes induced by BCM encoded cytokines. Therefore, we tested the effects of BCM and PCM on cytokine production in HKs. ELISA was used to confirm the production of cytokines IL-1β, IL-6, TNF-α, GM-CSF and chemokines CXCL-8 and CXCL-1 at the protein level. ELISA cytokine measurements at 4 and 24 hours were reported as picogram of cytokine per 100,000 adherent, non-apoptotic cells to account for the observed BCM-induced decrease in cell numbers and induction of apoptosis (Figure 4). ELISA data revealed that after four hours of treatment, BCM-treated HKs produced more cytokines, in agreement with the microarray data. After 24 hours of exposure to BCM, cytokines secreted by HKs leveled off, and in some cases, even decreased. After 24 hours, PCM-treated HKs produced roughly an order of magnitude more of each cytokine, with the exception of TNF-α, which was produced in greater quantities in BCM-treated HKs after 24 hours.

### S.*aureus *BCM suppresses JNK and p38 phosphorylation and induces MAPK independent cytokine production in human keratinocytes

**Figure 5:**
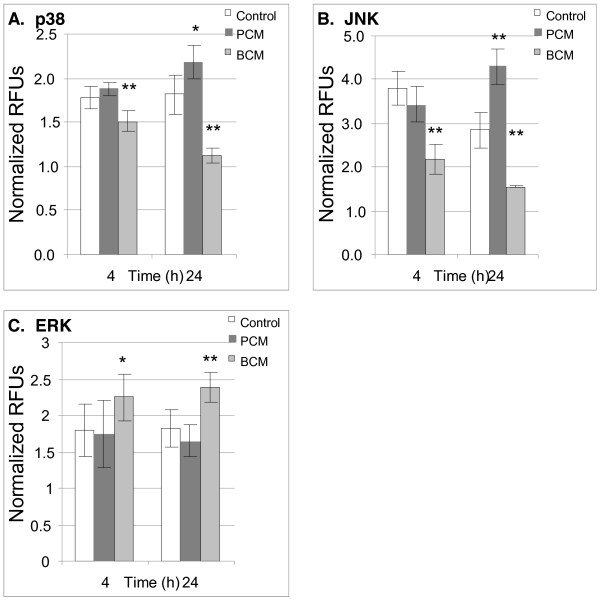
**MAPK phosphorylation in HKs exposed to BCM or PCM**. MAPK phosphorylation in HKs exposed to PCM or BCM for 4 or 24 hours. p38 (A) and JNK (B) phosphorylation levels were decreased in BCM treated HKs after 4 and 24 hours of exposure to BCM while PCM induced p38 and JNK phosphorylation after 24 hours. ERK phosphorylation (C) was unchanged in PCM treated HKs and increased in BCM treated HKs. Results represented as mean ± SD, n = 6, *p < 0.05, **p < 0.01 relative to control cells.

Functional enrichment of BCM induced genes revealed genes involved in MAPK cascades were over-represented in BCM treated HKs. To determine if the MAPKs JNK, p38, and ERK were differentially activated in HKs by BCM or PCM, levels of phosphorylated and total JNK, p38, and ERK were measured using cell-based ELISAs (Figure 5). Levels of phosphorylated JNK and p38 decreased after exposure to BCM. Exposure of HKs to PCM resulted in increased phosphorylation of JNK and to a lesser extent, p38. Phosphorylation of ERK was increased in BCM treated cells and unchanged in PCM treated cells. MAPK phosphorylation data were not normalized to adherent cell numbers as ratios of phosphorylated MAPK/total MAPK were measured only in adherent cells, accounting for reduced cell numbers. Apoptotic adherent cells were not accounted for in these data due to several reports of MAPK activation in apoptotic keratinocytes [22,23]. These data indicate that *S. aureus *BCM suppresses JNK and p38 phosphorylation levels below those of control cells which may lead to reduced cytokine levels.

**Figure 6:**
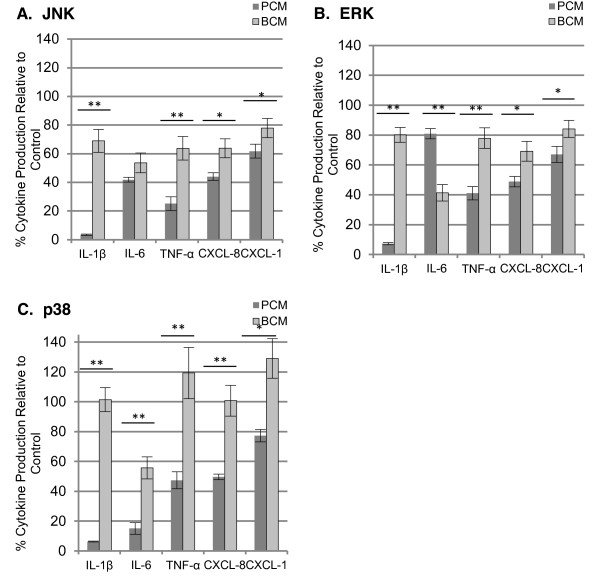
**MAPK inhibition and cytokine production in BCM and PCM treated HKs**. Cytokine production in MAPK inhibited HKs after 4 hours of exposure to PCM or BCM relative to non-inhibited control (BCM or PCM supplemented with DMSO). In general, MAPK inhibition resulted in a greater reduction of cytokine production in PCM treated HKs compared to BCM treated HKs. Results represented as mean ± SD, n = 3, *p < 0.05, **p < 0.01.

To investigate the effect of MAPK signaling on cytokine production in BCM and PCM-treated HKs, the MAPK family members JNK, p38, and ERK were inhibited using the inhibitors SP600125, SB203580, and U0126, respectively. Levels of GM-CSF were not analyzed in these experiments due to nearly undetectable levels at all time points except after 24 hours of exposure to PCM (Figure 4). Inhibition of JNK, p38, and ERK led to significant (p < 0.05) decreases in cytokine and chemokine production in PCM-treated HKs relative to BCM-treated HKs with the exception of IL-6 production in ERK-inhibited HKs (Figure 6). The data demonstrate that the majority of cytokines in BCM-treated HKs are produced through MAPK-independent mechanisms.

We have previously described characteristic morphology changes in BCM treated HKs [20]. The effects of MAPK inhibitors on BCM induced cell morphology were investigated here. Inhibition of JNK, p38, or ERK did not prevent the biofilm-induced formation of filopodial structures in HKs (data not shown). Overall, this indicates that cytoskeletal rearrangements induced by BCM act through MAPK-independent mechanisms.

## Discussion

*S. aureus *biofilm and planktonic-conditioned medium induced distinct responses in HKs *in vitro*. The adverse effects of planktonic bacterial cultures on mammalian cells have been well documented *in vitro*. Bacterial cells grown in broth cultures have long been assumed to retain the same pathogenic properties as bacteria in natural settings. While important discoveries have been realized based on planktonic studies, data presented here provide evidence that bacterial biofilms differentially induce pathogenesis in cultured HKs.

Host-pathogen interactions were investigated between a clinical isolate of *S. aureus *and HKs. A preliminary analysis of the extracellular proteome of *S. aureus *biofilm and planktonic cultures was performed by 1D gel electrophoresis and mass spectrometry. Several differences were observed in the 1D gel band patterns of BCM and PCM (Figure 1). The total protein concentrations of BCM and PCM were found to be similar, but BCM clearly contained more features. Smearing of BCM in 1D gels was observed indicating possible bacterial protease activity, although such a protease was not identified by mass spectrometry (Table 1). *S. aureus *secretes a variety of proteases which are important in pathogenesis [24]. The presence of such a protease could explain some of the observed effects in HKs after treatment with BCM or PCM.

Several 1D gel bands visible in PCM and not BCM contained glycolytic enzymes (Figure 1, Table 1). The presence of intracellular glycolytic enzymes in the extracellular proteome of *S. aureus *may be due to cell lysis, but cell wall associated glycolytic enzymes have been described for numerous pathogens, including *S. aureus *[25,26]. Links between central metabolism and virulence in *S. aureus *have been described. In *S. aureus*, when carbon sources are plentiful, glycolysis is active while the tricarboxcylic acid (TCA) cycle is largely repressed [27]. The TCA cycle has been described as a signal transduction pathway capable of regulating toxin production [28], adhesion synthesis and biofilm formation [29,30], and antibiotic susceptibility [31]. Additionally, *S. aureus *deletion mutants for the glycolytic enzymes gapA and gapB have been shown to have attenuated pathogenic capabilities [32]. The presence of several glycolytic enzymes in PCM and not in BCM supports the notion that central metabolic processes are in different states in planktonic and biofilm cultures and that those different metabolic states likely have a large impact on the observed pathogenic effects on HKs described here.

Functional annotation clustering of upregulated transcripts revealed over-represented annotation clusters associated with response to bacteria, regulation of transcription, inflammation, and signal transduction (Figure 2). The gene ontology term "response to glucocorticoid stimulus" was interesting as glucocorticoids are anti-inflammatory hormones. Genes involved in cyclic adenosine monophosphate (cAMP) signaling were also interesting since cAMP is involved in several fundamental cellular processes and may be partially responsible for the observed effects induced by BCM. Functional annotation clustering of downregulated transcripts revealed over-represented annotation clusters associated with transcription and metabolism. The downregulation of genes associated with these processes may indicate a general cessation in BCM treated cells.

Transcriptional responses of HKs to BCM revealed the upregulation of pro-inflammatory genes, including transcripts for pro-inflammatory transcription factors, cytokines, and apoptosis related genes. Among these were members of the AP-1 family of transcription factors and regulators of the NFkB pro-inflammatory transcription factor, TNFAIP3 (A20) and NFkBIA. Expression of these genes indicated active regulation of the NFkB pathway. NFkB regulates the expression of many genes involved in immune and inflammatory responses (i.e. cytokine and chemokine genes) and often acts in synergy with AP-1 to mediate inflammatory responses [33,34]. NFkB and AP-1 are activated by pro-inflammatory cytokines such as TNF-α and IL-1β which act through MAPK-dependent signal cascades resulting in the production of additional cytokines [35-38]. The transcription factor *egr1*, which was highly upregulated in BCM treated HKs, is also involved in the regulation of pathophysiologically important genes relating to inflammation, apoptosis, and differentiation [39-41]. The upregulation of these early response transcription factors indicates that four hours of treatment with BCM induces a swift inflammatory response in HKs relative to PCM.

We previously investigated BCM induced apoptosis and HK migration in a scratch wound model [20]. In agreement with that study, *S. aureus *BCM induced apoptosis in HKs while PCM did not induce a significant amount of apoptosis. BCM mediated induction of apoptosis is discussed in detail in [20]. This striking dissimilarity between PCM and BCM would undoubtedly have substantial impacts on several aspects of wound healing. Cytokine production induced by PCM and BCM were normalized to adherent non-apoptotic HKs.

ELISA analysis of cytokine production in HKs revealed that after four hours, BCM induced the production of more cytokines relative to PCM treated HKs. However, after 24 hours, BCM induced cytokine levels were weaker relative to cytokine production induced by PCM. Even though cytokine levels were normalized to non-apoptotic cells, it is important to note that early stage apoptosis may contribute to a general reduction in protein expression contributing to reduced cytokine levels. However, a reduction in MAPK phosphorylation indicates an alternative mechanism to early stage apoptosis for cytokine reduction.

Phosphorylation of the MAPKs JNK and p38 were found to be reduced by BCM while ERK was not. Inhibition of MAPK pathways revealed that MAPK signaling was responsible for a larger percentage of cytokine production in PCM treated HKs compared to BCM treated HKs. Even though there were strong differences in cytokine production between BCM and PCM treated cells after four hours, the representation of the inhibitor data as a percent of the vehicle control helps to reveal to what extent MAPKs are involved in cytokine production. SB203580, U0126, and SP600125 are widely used inhibitors of MAPKs. SB203580 and U0126 show a high degree of specificity towards p38 and ERK while the specificity of SP600125 towards JNK has recently been re-examined [42]. SP600125 was found to inhibit a wider range of kinases than initially thought. Given our goal to determine a generalized relationship between MAPK signaling and cytokine production, the reduced specificity of the JNK inhibitor SP600125 was tolerable. A specific role for p38, ERK, and JNK in *S. aureus *biofilm mediated host responses remains to be elucidated.

Several studies have investigated the inflammatory effects of planktonic bacterial supernatants on mammalian cells [43-52]. Genes upregulated by PCM were in agreement with the upregulation of pro-inflammatory genes in epithelial cells exposed to planktonic *S. aureus *supernatant [47]. Similar cytokine gene expression patterns were observed in human vaginal epithelial cells when exposed to late exponential phase *S. aureus *cultures [48]. Mid-logarithmic-phase cultures of *S. aureus *planktonic-conditioned medium induced IL-6, CXCL-8, and TNF-α in human-corneal-epithelial cells [44]. Different species of dental bacteria were found to induce various levels of the cytokines IL-1β, IL-6, and CXCL-8 after 4 or 24 hours of challenge in human gingival epithelial cells [52]; the ability of bacteria to induce cytokine production was correlated to the virulence of the strains tested.

Much less is known about the impacts of biofilm on mammalian cell cultures. *S. aureus *BCM initially induced higher levels of cytokines in HKs after four hours of exposure followed by reduced levels of cytokine production after 24 hours of exposure relative to PCM. The exception was TNF-α, which was found to be produced at higher levels in BCM treated HKs relative to PCM treated HKs. TNF-α is a cytokine capable of inducing apoptosis in many cell types including keratinocytes [53] and may be partially responsible for the observed increase in apoptotic HKs after exposure to BCM. In one *in vitro *host-pathogen model incorporating dental biofilms and human gingival epithelial cells, the cytokines IL-1β, IL-6 and CXCL-8 were degraded by the biofilm after four hours [54]. In that study, direct contact with the biofilm was required for biofilm mediated degradation of cytokines as filtered biofilm supernatant similar to BCM did not induce the degradation of cytokines. Our results showed that direct contact with the biofilm was not necessary for the observed decreases in cytokine production after 24 hours of exposure. A recent study investigating the effects of *S. aureus *biofilm infection in a mouse model found adaptive immune responses were regulated through cytokine production as the biofilm matured [55]. In that study, the production of key cytokines at certain times during the infection was hypothesized to manipulate the host's adaptive immune response resulting in localized tissue damage allowing *S. aureus *to establish a mature biofilm and mount a successful infection.

The patterns of cytokine and chemokine production from HKs exposed to either PCM or BCM are analogous to the patterns of cytokines produced during sepsis and chronic inflammatory diseases, respectively. Sepsis is characterized by release of massive amounts of cytokines and is analogous to the effects of PCM on cytokine production in HKs. Chronic inflammation, on the other hand, is similar to the effects of BCM where local inflammation is induced, but a runaway, self-inducing inflammatory response is not produced.

Three sub-types of MAPKs have been identified in mammals, ERK, JNK, and p38. JNK and p38 activation in HKs by PCM agree with other reports of JNK and p38 activation in mammalian cell cultures in response to bacterial cultures similar to the planktonic cultures described in this research [44,56-60]. Suppression of JNK and p38 phosphorylation in BCM-treated HKs below that of control and PCM-treated HKs occurred after 4 hours. Transcriptional analysis of BCM-treated HKs revealed the upregulation of dual specificity MAPK negative regulators, which may be responsible for the de-phosphorylation of JNK and p38 (Additional file 1). ERK is involved in the regulation of differentiation, apoptosis, and motility [61]. The activation of ERK may be associated with the regulation of these processes in HKs treated with BCM.

Chemical inhibition of MAPKs confirmed that PCM treatment induced more MAPK-dependent cytokine production than BCM in HKs after 4 hours of stimulation. The relative ineffectiveness of the MAPK inhibitors on BCM mediated cytokine production in addition to the reduced phosphorylation status of JNK and p38 suggests that BCM induces cytokine production through MAPK independent signaling mechanisms and the production of different factors by *S. aureus *biofilm compared to planktonic cultures.

The suppression of MAPK signaling by BCM could impact other wound-related activities involving MAPK cascades in HKs including HK differentiation [62], secretion of antimicrobial peptides [63], response to mechanical stress [64], and response to osmotic stress [65]. Suppression of MAPK signal transduction in HKs would be detrimental to all phases of wound healing, possibly contributing to the formation and/or persistence of chronic wounds. The observed upregulation of pro-inflammatory transcription factors at four hours may be an attempt by the cell to compensate for reduced MAPK signaling. The consequence of the overproduction of pro-inflammatory transcription factors could be the cause for the greater production of cytokines in BCM-treated HKs at four hours. Several transcription factors are differentially regulated in BCM treated HKs. Certain transcription factors induce or inhibit AP-1. One such transcription factor is A20 which is known to activate AP-1 and inhibit activation of JNK [66]. A20 was upregulated 3.09 fold in BCM treated HKs relative to PCM treated cells (Additional file 1). It is possible that other MAPK independent pathways are activated or inhibited by BCM mediated MAPK inactivation resulting in A20 expression, leading to the initial increase of AP-1 family transcription factors.

Guggenheim et al. found that cytokines were degraded by direct contact with an *in vitro *dental biofilm [54]. The smearing of BCM proteins on 1D gels indicates the possible presence of a *S. aureus *protease that may be responsible for the degradation of excreted cytokines. However, the suppression of MAPK phosphorylation and MAPK independent production of cytokines in BCM treated HKs suggests that cytokine production is at least partially limited through this important signaling pathway. MAPK suppression in various mammalian cell types by bacterial toxins has been observed. *Bacillus anthracis *secretes lethal toxin, which cleaves most isoforms of MAPKs, reducing pro-inflammatory cytokine secretion from immune cells [67]. *Shigella flexneri, Yersinia *spp., and *Salmonella *spp. deliver toxins which inhibit MAPK signal transduction through a type III secretion mechanism resulting in the repression of genes such as TNF-α, IL-6, and CXCL-8 [68,69]. To our knowledge, a toxin has not been identified in *S. aureus *that inhibits MAPK signaling, but it is tempting to speculate that such a toxin exists and is responsible for the observed suppression of p38 and JNK phosphorylation. The results presented here provide the basis to characterize the response of HKs to BCM and allow the formulation and testing of hypotheses as to specific components in BCM that cause the observed HK response. Metabolomic and proteomic characterization of BCM are beyond the scope of the present work, but it is relevant to mention that preliminary MS and NMR-based metabolomics analysis revealed numerous metabolites specific to *S. aureus *BCM (Our unpublished observations).

A hypothetical mechanism of pathogenesis induced by *S. aureus *infection as related to this work is presented here. The initial infection of wounded tissue is assumed to be primarily by planktonic *S. aureus*. That infection could result in a normal inflammatory response where the invading bacteria are destroyed and the tissue progresses through a normal healing response. If the host were immune-compromised, had an underlying disease (i.e. diabetes, pulmonary disease, or other inflammatory diseases), or conditions were favorable for the pathogen, *S. aureus *could successfully evade the immune system. If *S. aureus *were successful in evading the host's immune response, the resulting infection could continue to spread, reach the bloodstream and induce sepsis, resulting in death (i.e. a planktonic *S. aureus *infection). Alternatively, *S. aureus *could revert to a biofilm growth phase where HK apoptosis and cytoskeletal rearrangements would inhibit the re-epithelialization of the wound [20] and a deranged inflammatory response could establish a localized, persistent infection.

## Conclusions

These data provide insights into mechanisms of pathogenesis in biofilm-based chronic-wound infections. Processes relating to epithelialization such as the disruption of cytoskeletal components and induction of apoptosis are induced by BCM in HKs. Suppression of MAPK signaling and the corresponding derangement of cytokine production in BCM treated HKs could help to explain the local, chronic inflammation observed in biofilm-infected skin. Analysis of the extracellular proteome of *S. aureus *suggested that planktonic and biofilm cultures were in different metabolic states which may impact pathogenesis in HKs. Collectively, the results help explain the formation and persistence of chronic wounds. Additionally, the differences in pathogenesis between bacterial biofilm and planktonic cultures detailed here highlight the importance of considering biofilm formation in any model of disease.

## Methods

### Cell Culture

Human foreskin keratinocytes (HFKs) and the spontaneously immortalized human HaCaT keratinocyte cell line were used. HaCaT keratinocytes are a widely used keratinocyte line which displays similar responses to TLR ligands as primary keratinocytes and is suitable for studies investigating innate immunity [14]. Additionally, HaCaT keratinocytes undergo the same BCM induced morphology changes, induction of apoptosis, and increases in intracellular calcium as HFKs (this study and our unpublished observations).

HFKs were cultured from newborn foreskin and passaged in serum free medium using methods previously described [70]. Cells were maintained in EpiLife^® ^keratinocyte growth medium (Cascade Biologics, Portland, OR) supplemented with human keratinocyte growth supplement (HKGS; Cascade Biologics, Portland, OR). Experiments were conducted with cell passages 4-10, using EpiLife^® ^medium supplemented with HKGS (EPI). HaCaT keratinocytes were maintained under identical conditions. All cultures were kept in a humidified 5% CO_2 _incubator at 37°C.

### *S. aureus *Biofilm Culture Conditions and Preparation of BCM

Tissue culture inserts (35 mm diameter, 0.2 μm pore size, Nalge Nunc International, Rochester, NY) were placed into six well plates with 2.1 ml of EPI in each well. An initial overnight culture of a clinical isolate of *S. aureus *(Southwest Regional Wound Care isolate # 10943, Lubbock, TX) was diluted in EPI to an optical density of 0.05 at 600 nm. Seven 10 μl drops of the diluted overnight culture were placed onto individual culture inserts and biofilms were allowed to develop and mature for 72 hours. Every 24 hours for four days thereafter, the growth medium was collected, filter sterilized, pH adjusted to 7.2, and replaced with fresh EPI. The collected medium is referred to as BCM. *S. aureus *BCM was pooled to provide sufficient quantities of material to work with and to help eliminate day to day variations that might occur in the biofilm cultures.

### Planktonic *S. aureus *Culture Conditions and Preparation of PCM

Planktonic *S. aureus *cultures were grown under conditions designed to produce similar cell densities and physiology (i.e. stationary phase growth) as the biofilm cultures. To obtain such a culture, mature three day old biofilms grown on tissue culture inserts were re-suspended into the same volume of EPI growth medium in which biofilm cultures were maintained and cultured at 37°C with constant agitation. This method effectively reverted *S. aureus *cells from biofilm growth back to planktonic growth. Planktonic bacteria were removed from solution by centrifugation. The supernatant was collected, filter sterilized, and pH adjusted to 7.2. The bacterial pellet was resuspended in EPI and cultured at 37°C with constant agitation for an additional 24 hours. This process was repeated every 24 hours for four days and the conditioned medium pooled to provide sufficient material to work with and to help eliminate day to day variations that might occur in overnight planktonic cultures. The pooled, sterilized supernatant is referred to as PCM. Both planktonic and re-suspended biofilm cultures of *S. aureus *contained similar population densities based on optical density (600 nm) readings at 4 and 24 hours.

### SDS-PAGE analysis and in-gel digestion for protein identification

Total protein from BCM, PCM, and EpiLife growth medium was quantified using a modified Lowry assay following the manufacturer's protocol (Thermo Scientific, Rockford, IL). Proteins were precipitated from 2 ml of sample by adding 200 μl of a 1:4 solution of trichloroacetic acid and acetone. The solution was incubated at 4°C for an hour. Samples were then centrifuged at 14,000 rpm for 15 minutes at 4°C. The supernatant was decanted and the pellet was washed with 500 μl cold acetone and centrifuged. After removing the supernatant, protein pellets were dried at room temperature and re-suspended in 30 μl sample buffer (3.8 ml water, 1 ml 0.5 M Tris-HCl, pH 6.8, 0.8 ml glycerol, 1.6 ml 10% SDS, 0.4 ml 2-β-mercaptoethanol, 0.4 ml 0.05% (W/V) bromophenol blue). Samples were incubated at 95°C for 5 minutes. Samples were run on a 12% acrylamide gel and stained with Coomassie brilliant blue R250 (BioRad, Hercules, CA). Excised gel slices were destained using 50% acetonitrile in 50 mM ammonium bicarbonate (pH 7.9) and vacuum dried. Samples were rehydrated with 1.5 mg/ml dithiothreitol (DTT) in 25 mM ammonium bicarbonate (pH 8.5) at 56°C for 1 h, subsequently alkylated with 10 mg/ml iodoacetamide (IAA) in 25 mM ammonium bicarbonate (pH 8.5), and stored in the dark at room temperature for 1 h. The pieces were subsequently washed with 100 mM ammonium bicarbonate (pH 8.5) for 15 min, washed twice with 50% acetonitrile in 50 mM ammonium bicarbonate (pH 8.5) for 15 min each, vacuum dried, and rehydrated with 4 μl of proteomics grade modified trypsin (100 μg/ml; Sigma, St. Louis, MO) in 25 mM ammonium bicarbonate (pH 8.5). The pieces were covered in a solution of 10 mM ammonium bicarbonate with 10% acetonitrile (pH 8.5) and incubated at 37°C for 16 h.

### Liquid Chromatography-Tandem Mass Spectrometry

Liquid chromatography coupled to tandem mass spectrometry (LC/MS-MS) analysis was conducted at the Mass Spectrometry Laboratory at Montana State University. Peptides were separated on a microfluidic ChipCube interface and detected with an ESI-Trap XCT Ultra instrument (Agilent, Santa Clara, CA). The MASCOT search engine was used to compare peptide masses determined by MS to masses of sequences in the NCBInr bacterial database. Acceptable protein identifications required expectation values of 0.01 for LC-MS/MS.

### Microarray

HFKs were grown to 90% confluence in six well plates. Cells were then treated with 2 ml BCM, PCM, or EPI for four hours. After treatment, the medium was removed and RNA was isolated using an RNeasy minikit (Qiagen, Valencia, CA) following the manufacturer's instructions for adherent cells. Extracted RNA was ethanol precipitated and resuspended in water as previously described [71]. RNA concentrations and purity were determined by measuring absorbencies at 260 nm and 280 nm on a GeneQuant spectrophotometer. RNA quality was also evaluated using the RNA 6000 NanoChip assay on a 2100 Bioalyzer (Agilent Technologies, Palo Alto, CA) in the Functional Genomics Core Facility at Montana State University. RNA integrity number for all samples used exceeded 9.5 on a scale to 10.

Total RNA (500 ng) was reverse transcribed, amplified and biotin-labeled via *in vitro *transcription using the MessageAmp Premier kit (Applied Biosystems/Ambion, Austin, TX). The resulting cRNA was fragmented and hybridized to Affymetrix GeneChip Human Genome U133A 2.0 arrays (#900468, Affymetrix, Santa Clara, CA) at 45°C for 16 hours with constant rotational mixing at 60 rpm. Washing and staining of the arrays was performed using the Affymetrix GeneChip Fluidics Station 450. Arrays were scanned using an Affymetrix GeneChip Scanner 7G and GCOS software version 1.4.

Microarray data were analyzed using FlexArray version 1.4. The Affymetrix CEL files were imported and normalized using GC-RMA. Genes were filtered for threshold signal intensities of at least 50 in one biological replicate. Analysis of Variance (ANOVA) was performed to identify statistically significant differences among the three conditions. 910 genes were identified (p-value < 0.01). The gene list was further trimmed to identify genes with fold-change differences of at least 1.5 in any comparison, resulting in 575 genes. The log2 values were imported into Genesis [72] for visualization and hierarchical clustering. Data were submitted to Gene Expression Omnibus (NCBI) under accession GSE24118. Subsequent functional enrichment analysis was conducted using the database for annotation, visualization and integrated discovery (DAVID) software [73]. The functional annotation clustering tool was used to identify over-represented gene ontology terms (p < 0.05; Benjamini correction for multiple testing) with the conservative high stringency option. Significantly upregulated or downregulated genes with a fold change ± 1.5 (BCM relative to PCM) were submitted as separate lists. Functional annotation clusters with an enrichment score greater than 1.5 were considered significant.

### Cytokine Detection by ELISA

Confluent HaCaT keratinocytes in 6-well plates were cultured in the presence of bacterial conditioned medium (BCM or PCM) for 4 or 24 hours. Cell culture supernatants were collected and analyzed by colorimetric sandwich enzyme-linked immunoassays (ELISA) for IL-1β, IL-6, TNF-α, CXCL-8, CXCL-1, and GM-CSF (R&D Systems, Minneapolis, MN) following the manufacturer's instructions. Cytokines in the supernatant were detected as pg/ml. HKs remaining in the culture wells were stained with propidium iodide and counted. Cell counts per well and the measured percentage of pro-apoptotic cells revealed by Terminal Deoxynucleotidyl Transferase dUTP Nick End Labeling **(**TUNEL) were used to normalize ELISA data to pg/100,000 adherent, non-apoptotic cells.

### Detection of MAPK Phosphorylation

HaCaT keratinocytes were grown to confluence in clear bottom black walled 96-well plates. Keratinocytes were treated with BCM or PCM for 4 or 24 hours. Total and phosphorylated MAPKs (JNK, p38, and ERK) were detected simultaneously using a cell-based ELISA (R&D Systems, Minneapolis, MN) following the manufacturer's instructions.

### Inhibition of MAPK

The p38 MAPK inhibitor, SB203580; the ERK inhibitor, U0126; and the JNK inhibitor, SP600125 were prepared as 10 mM DMSO stocks (Cayman Chemicals, Ann Arbor, MI). Confluent HaCaT keratinocytes were pretreated with individual inhibitors or a combination of all three inhibitors (10 μM each, 0.1% DMSO) in EPI growth medium for one hour. Cells were then treated with PCM or BCM supplemented with 10 μM inhibitor(s) for four hours. Cell culture supernatants were collected and analyzed by ELISA for cytokine production. HaCaT keratinocytes treated with PCM or BCM supplemented with 0.1% DMSO were prepared as vehicle controls.

### Detection of apoptosis by TUNEL

TUNEL staining was used to investigate the induction of apoptosis. HaCaT keratinocytes were grown to 90% confluence on 18 mm^2 ^glass cover slips placed in six-well plates. Keratinocytes were then exposed to 2 ml BCM, PCM, or EPI. At 4 or 24 hours, apoptotic keratinocytes were detected using the APO-BrdU TUNEL Assay Kit (Invitrogen, Carlsbad, CA) following the manufacturer's staining protocol as previously described. Cells were counter stained with propidium iodide. Coverslips were imaged using a Nikon Eclipse E800 epifluorescent microscope using a 10 × objective. For analysis, four images of each condition were taken and numbers of adherent cells staining positive for TUNEL and propidium iodide were counted and the percentage of cells staining positive for TUNEL were calculated.

## List of abbreviations

BCM: biofilm-conditioned medium; PCM: planktonic conditioned medium; MAPK: mitogen activated protein kinase; PG: peptidoglycan; LTA: lipoteichoic acid; HKs: human keratinocytes; PAMPs: pathogen associated molecular patterns; TLR: toll like receptor; AP-1: activated protein 1; NFkB: nuclear factor kappa B; TUNEL: terminal deoxynucleotidyl transferase dUTP nick end labeling.

## Competing interests

The authors declare that they have no competing interests.

## Authors' contributions

PRS was responsible for culturing keratinocytes and *S. aureus*, SDS-PAGE analysis, ELISA assays, MAPK analysis, running TUNEL assays, RNA extractions, and drafted the manuscript. KM carried out microarray sample processing and analysis. GAJ, PF, JEO, and PSS conceived of the study, participated in its design and coordination, and helped to draft the manuscript. All authors read and approved the final manuscript.

## Supplementary Material

Additional file 1 **Genes significantly regulated in BCM treated HKs**. Transcriptional profile (fold change ±1.5, pval < 0.01 BCM relative to PCM) of HKs after four hours of exposure. Click here for file
