# Supplementary material for: Staphylococcus aureus Biofilm and Planktonic cultures differentially impact gene expression, mapk phosphorylation, and cytokine production in human keratinocytes
Source: BMC Microbiol. 2011 Jun 21;11:143. doi: 10.1186/1471-2180-11-143 (PMC3146417; doi:10.1186/1471-2180-11-143)
Supplement: Additional file 1 — Genes significantly regulated in BCM treated HKs. Transcriptional profile (fold change ±1.5, pval < 0.01 BCM relative to PCM) of HKs after four hours of exposure. [file 1471-2180-11-143-S1.PDF]

Table 1. Transcriptional profile (fold change ±1.5 BCM relative to PCM) of HKs after four hours of exposure.

Cytokine Transcription Factor

Rank	Gene Name	Gene Symbol	Fold Change (4 h) PCM v control	BCM v Control	BCM v PCM	p-value
1	c-fos FBJ murine osteosarcoma viral oncogene homolog (AP-1)	c-FOS	1.01	43.58	42.97	2.59E-03
2	Early growth response 1	EGR1	-1.10	17.79	19.58	4.80E-03
3	Chemokine (C-X-C motif) ligand 8/Interleukin 8	CXCL-8/IL-8	6.14	64.43	10.49	4.01E-07
4	Chemokine (C-X-C motif) ligand 2	CXCL-2	6.21	62.11	10.00	3.08E-07
5	Activating transcription factor 3 (AP-1)	ATF3	1.42	13.63	9.61	1.06E-03
6	Basic helix-loop-helix family, member e40	BHLHE40	1.44	13.58	9.46	1.40E-03
7	Tumor necrosis factor alpha	TNF-α	3.05	27.73	9.10	4.00E-05
8	Hairy and enhancer of split 1 (Drosophila)	HES1	1.80	13.71	7.62	8.00E-05
9	Chemokine (C-X-C motif) ligand 3	CXCL-3	1.82	12.23	6.72	6.65E-04
10	Dual specificity phosphatase 1	DUSP1	1.62	10.13	6.24	4.82E-04
11	Zinc finger protein 36, C3H type, homolog (mouse)	ZFP36	1.76	7.08	4.02	4.99E-05
12	Interleukin 6	IL-6	1.06	3.40	3.20	1.79E-03
13	Tumor necrosis factor alpha-induced protein 3	TNFAIP3	4.39	13.59	3.10	1.06E-07
14	GTP binding protein overexpressed in skeletal muscle	GEM	5.93	18.02	3.04	1.28E-05
15	Histone cluster 2, H2aa3	HIST2H2AA3	-1.01	2.95	2.99	6.43E-03
16	Cyclin L1	CCNL1	1.03	2.86	2.78	7.92E-03
17	Chromosome 1 open reading frame 63	C1orf63	1.34	3.61	2.70	1.14E-03
18	Protein phosphatase 1, regulatory (inhibitor) subunit 15A	PPP1R15A	3.42	8.87	2.59	7.47E-05
19	Growth arrest and DNA-damage-inducible, beta	GADD45B	-1.06	2.30	2.44	6.74E-03
20	Cytochrome P450, family 1, subfamily A, polypeptide 1	CYP1A1	1.12	2.63	2.35	9.05E-03
21	Splicing factor proline/glutamine-rich	SFPQ	1.51	3.52	2.34	1.74E-04
22	S100 calcium binding protein P	S100P	1.29	2.86	2.21	1.98E-03
23	H2B histone family, member S	H2BFS	1.06	2.32	2.19	4.65E-03
24	Growth arrest and DNA-damage-inducible, beta	GADD45B	1.01	2.18	2.15	7.18E-03
25	Vascular endothelial growth factor A	VEGFA	3.93	8.08	2.05	1.12E-04
26	Jun oncogene (AP-1)	c-JUN	1.78	3.64	2.05	3.54E-05
27	Syntaxin 5	STX5	1.04	1.93	1.86	4.89E-03
28	ER degradation enhancer, mannosidase alpha-like 2	EDEM2	1.13	2.09	1.86	6.00E-03
29	Colony stimulating factor 3 (granulocyte)	CSF3/G-CSF	1.54	2.84	1.84	6.73E-05
30	EPS8-like 2	EPS8L2	1.11	2.05	1.84	3.40E-03
31	Cyclin-dependent kinase 2 associated protein 2	CDK2AP2	1.03	1.89	1.83	6.25E-03
32	Histone cluster 1, H2bd	HIST1H2BD	1.30	2.37	1.82	9.86E-03
33	RanBP-type and C3HC4-type zinc finger containing 1	RBCK1	1.05	1.84	1.76	4.46E-03
34	Colony stimulating factor 2 (granulocyte-macrophage)	CSF2/GM-CSF	2.43	4.24	1.75	5.36E-04
35	Glutathione S-transferase mu 1	GSTM1	1.08	1.87	1.73	1.16E-03
36	Chromosome 9 open reading frame 95	C9orf95	1.25	2.12	1.69	5.25E-03
37	Serine/threonine/tyrosine interacting-like 1	STYXL1	1.18	1.98	1.68	3.26E-03
38	Vacuolar proton-ATPase subunit M9.2	ATP6V0E1	1.07	1.81	1.68	9.09E-03
39	Ephrin-A1	EFNA1	3.84	6.44	1.68	1.59E-04
40	6-phosphogluconolactonase	PGLS	-1.04	1.61	1.67	8.60E-03
41	Pleckstrin homology-like domain, family A, member 1	PHLDA1	1.13	1.88	1.66	4.33E-03
42	Hexosaminidase A (alpha polypeptide)	HEXA	1.09	1.81	1.65	9.81E-03
43	Anoctamin 10	ANO10	-1.05	1.56	1.64	9.14E-03
44	OTU domain, ubiquitin aldehyde binding 1	OTUB1	1.14	1.86	1.64	6.04E-03
45	Glutathione S-transferase mu 2 (muscle)	GSTM2	1.16	1.88	1.62	2.98E-03
46	ECSIT homolog (Drosophila)	ECSIT	1.19	1.92	1.62	3.26E-04
47	Small nuclear ribonucleoprotein polypeptide A	SNRPA	-1.00	1.61	1.61	9.27E-03
48	Histone cluster 1, H2bk	HIST1H2BK	1.03	1.64	1.59	8.52E-03
49	Prolyl 4-hydroxylase, alpha polypeptide I	P4HA1	1.23	1.95	1.59	5.60E-03
50	Jun B proto-oncogene (AP-1)	JUNB	2.31	3.65	1.58	1.48E-05
51	Insulin-like growth factor 2 (somatomedin A)	IGF2	1.46	2.31	1.58	2.11E-04
52	Chromosome 9 open reading frame 16	C9orf16	1.18	1.86	1.58	6.34E-04
53	Neuron derived neurotrophic factor	NENF	1.03	1.63	1.58	6.15E-03
54	TP53 target 1 (non-protein coding)	TP53TG1	1.07	1.68	1.58	8.16E-03
55	Collagen, type I, alpha 2	COL1A2	1.08	1.70	1.57	1.30E-03
56	Heparan sulfate proteoglycan 2	HSPG2	1.13	1.78	1.57	4.02E-03
57	Tetraspanin 4	TSPAN4	1.02	1.60	1.56	2.51E-03
58	Nuclear factor of kappa light polypeptide gene enhancer inhibitor α	NFKBIA	2.35	3.65	1.55	1.17E-04
59	Fatty acid desaturase 1	FADS1	1.02	1.58	1.55	3.99E-03
60	Zinc finger, DHHC-type containing 24	ZDHHC24	1.11	1.72	1.54	8.63E-03
61	Fas-activated serine/threonine kinase	FASTK	1.02	1.57	1.54	5.53E-03
62	Coatomer protein complex, subunit zeta 2	COPZ2	1.08	1.63	1.52	1.93E-03
63	Annexin A6	ANXA6	1.07	1.61	1.51	8.45E-03
64	Chemokine (C-X-C motif) ligand 1	CXCL-1	13.86	20.81	1.50	5.35E-04
65	Cyclin B1 interacting protein 1	CCNB1IP1	1.04	1.56	1.50	6.72E-03
66	Eukaryotic translation initiation factor 4 gamma, 1	EIF4G1	-1.11	-1.67	-1.51	5.12E-04

67	Zinc finger, matrin type 3	ZMAT3	-1.04	-1.57	-1.51	3.01E-03
68	Vacuolar protein sorting 54 homolog (S. cerevisiae)	VPS54	-2.09	-3.15	-1.51	3.64E-03
69	Protein phosphatase 4, regulatory subunit 1	PPP4R1	-1.14	-1.73	-1.51	2.85E-03
70	Microfibrillar-associated protein 1	MFAP1	-1.27	-1.92	-1.51	6.93E-03
71	HLA-B associated transcript 2	BAT2	-1.18	-1.78	-1.51	8.22E-04
72	Mediator complex subunit 6	MED6	-1.33	-2.01	-1.51	7.22E-05
73	Uridine monophosphate synthetase	UMPS	-2.41	-3.65	-1.51	2.15E-04
74	Solute carrier family 4, member 7	SLC4A7	-1.48	-2.24	-1.51	7.34E-05
75	SHC SH2-domain binding protein 1	SHCBP1	-1.95	-2.95	-1.51	8.11E-03
76	Zinc finger, MYND domain containing 11	ZMYND11	-1.32	-1.99	-1.52	6.07E-03
77	RAB1A, member RAS oncogene family	RAB1A	-1.16	-1.76	-1.52	1.58E-03
78	Translocase of outer mitochondrial membrane 70 homolog A	TOMM70A	-1.20	-1.83	-1.52	1.44E-03
79	Interleukin 1 receptor accessory protein	IL1RAP	-1.40	-2.13	-1.52	6.90E-04
80	Zinc finger, BED-type containing 1	ZBED1	-1.31	-2.00	-1.52	1.03E-04
81	DEAD (Asp-Glu-Ala-Asp) box polypeptide 28	DDX28	-2.01	-3.07	-1.52	8.17E-05
82	Plectin 1, intermediate filament binding protein 500kDa	PLEC1	-1.21	-1.84	-1.52	3.74E-03
83	UTP3, small subunit (SSU) processome component	UTP3	-1.68	-2.57	-1.53	1.19E-05
84	Rho guanine nucleotide exchange factor (GEF) 3	ARHGEF3	-1.26	-1.92	-1.53	2.40E-03
85	TATA element modulatory factor 1	TMF1	-1.35	-2.06	-1.53	2.17E-03
86	Signal-induced proliferation-associated 1 like 3	SIPA1L3	-1.11	-1.70	-1.53	1.88E-03
87	RAN binding protein 2	RANBP2	-1.44	-2.21	-1.54	7.93E-04
88	Family with sequence similarity 168, member B	FAM168B	1.00	-1.53	-1.54	5.93E-03
89	CAP-GLY domain containing linker protein 1 (restin)	CLIP1	-1.27	-1.96	-1.54	1.04E-03
90	PTK2 protein tyrosine kinase 2	PTK2	-1.08	-1.66	-1.54	2.54E-03
91	Zinc finger, MYND-type containing 8	ZMYND8	-2.26	-3.48	-1.54	2.37E-03
92	RNA binding motif protein 15B	RBM15B	-1.30	-2.00	-1.54	3.56E-03
93	F-box protein 42	FBXO42	-1.27	-1.96	-1.54	3.22E-03
94	Transcription elongation factor B (SIII), polypeptide 3	TCEB3	-2.50	-3.87	-1.55	2.21E-04
95	Zinc finger protein 329	ZNF329	-1.62	-2.52	-1.55	6.54E-03
96	Calcium homeostasis endoplasmic reticulum protein	CHERP	-1.79	-2.77	-1.55	3.80E-04
97	Filamin B, beta (actin binding protein 278)	FLNB	-1.02	-1.59	-1.56	7.53E-03
98	Transcription factor A, mitochondrial	TFAM	-1.74	-2.72	-1.56	3.55E-03
99	GTPase activating Rap/RanGAP domain-like 4	GARNL4	-1.23	-1.93	-1.57	1.09E-03
100	Integrator complex subunit 5	INTS5	-1.89	-2.97	-1.57	2.57E-03
101	Inhibitor of kappa light polypeptide gene enhancer in B-cells	IKBKAP	-1.25	-1.97	-1.57	6.82E-04
102	Chromosome 19 open reading frame 2	C19orf2	-1.27	-1.99	-1.57	1.45E-03
103	CCR4-NOT transcription complex, subunit 1	CNOT1	-1.37	-2.15	-1.57	5.72E-04
104	Excision repair cross-complementing rodent repair deficiency	ERCC6L	-2.00	-3.15	-1.57	7.17E-05
105	Heterogeneous nuclear ribonucleoprotein H3 (2H9)	HNRNPH3	-1.09	-1.71	-1.58	7.80E-03
106	Mitogen-activated protein kinase 6	MAPK6 (erk 3)	-1.23	-1.95	-1.58	3.46E-03
107	KIAA1219	KIAA1219	-1.59	-2.51	-1.58	4.05E-03
108	Golgi SNAP receptor complex member 1	GOSR1	-1.68	-2.65	-1.58	1.29E-05
109	F-box and WD repeat domain containing 2	FBXW2	-1.19	-1.88	-1.58	9.92E-03
110	PR domain containing 4	PRDM4	-1.14	-1.80	-1.58	3.34E-03
111	Heterogeneous nuclear ribonucleoprotein H3 (2H9)	HNRNPH3	-1.14	-1.80	-1.58	2.15E-03
112	Sec23 homolog B (S. cerevisiae)	SEC23B	-1.48	-2.34	-1.58	5.39E-03
113	Suppressor of Ty 6 homolog (S. cerevisiae)	SUPT6H	-1.05	-1.68	-1.59	5.02E-03
114	Dpy-19-like 1 (C. elegans)	DPY19L1	-1.11	-1.78	-1.60	3.01E-03
115	Zinc finger with KRAB and SCAN domains 1	ZKSCAN1	-2.09	-3.35	-1.60	3.61E-03
116	CCCTC-binding factor (zinc finger protein)	CTCF	-2.57	-4.13	-1.60	1.66E-03
117	Protein tyrosine phosphatase, non-receptor type 11	PTPN11	-1.06	-1.70	-1.61	7.64E-03
118	F-box protein 28	FBXO28	-1.39	-2.24	-1.61	7.44E-03
119	Protein tyrosine phosphatase type IVA, member 1	PTP4A1	-1.02	-1.65	-1.61	4.17E-03
120	Histone acetyltransferase 1	HAT1	-1.11	-1.79	-1.61	1.13E-03
121	Aspartate beta-hydroxylase	ASPH	-1.25	-2.02	-1.61	2.29E-05
122	Dual specificity phosphatase 11 (RNA/RNP complex 1-interacting)	DUSP11	-1.56	-2.53	-1.62	1.49E-05
123	A kinase (PRKA) anchor protein 11	AKAP11	-1.60	-2.59	-1.62	4.89E-03
124	Structural maintenance of chromosomes 5	SMC5	-1.37	-2.21	-1.62	9.34E-03
125	CCAAT/enhancer binding protein (C/EBP), zeta	CEBPZ	-1.49	-2.43	-1.63	7.48E-04
126	Cyclin T2	CCNT2	-1.84	-2.99	-1.63	5.17E-04
127	Jumonji, AT rich interactive domain 2	JARID2	-1.21	-1.97	-1.64	3.42E-04
128	SRY (sex determining region Y)-box 4	SOX4	-1.40	-2.29	-1.64	4.07E-04
129	Kinesin light chain 1	KLC1	-1.22	-2.00	-1.65	4.03E-03
130	Absent in melanoma 1	AIM1	-1.08	-1.79	-1.65	1.49E-03
131	Sideroflexin 1	SFXN1	-1.13	-1.88	-1.66	4.04E-03
132	Mannosyl glycoprotein beta-1,2-N-acetylglucosaminyltransferase	MGAT2	-1.71	-2.84	-1.66	1.16E-03
133	Nucleoporin 62kDa	NUP62	-1.25	-2.08	-1.67	1.61E-03
134	PDZ binding kinase	PBK	-1.32	-2.20	-1.67	1.70E-03
135	C2 calcium-dependent domain containing 2	C2CD2	-1.64	-2.74	-1.67	7.20E-04
136	KIAA1279	KIAA1279	-1.71	-2.87	-1.67	1.45E-03
137	Cullin 3	CUL3	-2.59	-4.34	-1.68	9.25E-04
138	Centromere protein A	CENPA	-1.30	-2.20	-1.69	6.16E-04

139	Tumor protein p63	TP63	-1.15	-1.95	-1.70	5.51E-04
140	TATA box binding protein associated factor, RNA pol I, B, 63kDa	TAF1B	-1.16	-1.98	-1.71	6.29E-03
141	PR domain containing 4	PRDM4	1.01	-1.70	-1.71	8.36E-03
142	Ring finger protein 34	RNF34	-1.66	-2.84	-1.71	3.66E-03
143	Leucine rich repeat (in FLII) interacting protein 1	LRRFIP1	-1.04	-1.78	-1.72	4.08E-03
144	Kinesin family member 20B	KIF20B	-1.28	-2.20	-1.72	6.21E-04
145	Tetraspanin 3	TSPAN3	1.01	-1.70	-1.72	6.59E-03
146	Vitamin D (1,25- dihydroxyvitamin D3) receptor	VDR	-1.52	-2.62	-1.73	3.46E-06
147	Ectonucleoside triphosphate diphosphohydrolase 4	ENTPD4	-2.05	-3.55	-1.74	7.39E-03
148	Pogo transposable element with KRAB domain	POGK	-2.28	-3.96	-1.74	4.67E-06
149	Squalene epoxidase	SQLE	-1.01	-1.76	-1.74	3.02E-03
150	Ribosomal RNA processing 1 homolog B (S. cerevisiae)	RRP1B	-1.18	-2.07	-1.75	2.14E-03
151	E1A binding protein p400	EP400	-1.28	-2.23	-1.75	5.11E-05
152	X-ray repair complementing defective repair 4	XRCC4	-1.39	-2.43	-1.75	9.61E-05
153	Cysteine rich transmembrane BMP regulator 1 (chordin-like)	CRIM1	-1.05	-1.84	-1.75	8.46E-03
154	YLP motif containing 1	YLPM1	-1.22	-2.14	-1.75	5.06E-03
155	Thrombomodulin	THBD	-2.17	-3.82	-1.75	3.56E-05
156	Eukaryotic translation initiation factor 3, subunit A	EIF3A	-2.43	-4.28	-1.76	4.26E-03
157	Protocadherin 7	PCDH7	-2.10	-3.70	-1.76	2.60E-04
158	DEAH (Asp-Glu-Ala-His) box polypeptide 15	DHX15	1.01	-1.75	-1.77	6.63E-03
159	YTH domain family, member 2	YTHDF2	-1.31	-2.31	-1.77	1.14E-03
160	Basonuclin 1	BNC1	-1.04	-1.84	-1.77	6.41E-03
161	Poly (ADP-ribose) polymerase 1	PARP1	1.02	-1.75	-1.77	6.49E-03
162	Neuroguidin, EIF4E binding protein	NGDN	-1.49	-2.65	-1.78	4.20E-03
163	Family with sequence similarity 3, member C	FAM3C	-1.13	-2.03	-1.79	8.51E-03
164	Protocadherin 7	PCDH7	-1.69	-3.03	-1.79	8.54E-04
165	Myeloid cell leukemia sequence 1 (BCL2-related)	MCL1	-1.12	-2.00	-1.79	2.53E-03
166	Discs, large homolog 5 (Drosophila)	DLG5	-1.05	-1.89	-1.80	2.01E-03
167	MOB1, Mps One Binder kinase activator-like 3 (yeast)	MOBKL3	-1.50	-2.70	-1.80	5.01E-03
168	Adenosine deaminase, RNA-specific	ADAR	-1.03	-1.87	-1.81	6.62E-03
169	Dicer 1, ribonuclease type III	DICER1	-2.93	-5.31	-1.81	2.59E-04
170	FYVE and coiled-coil domain containing 1	FYCO1	-1.31	-2.38	-1.81	1.17E-03
171	Vitamin D (1,25- dihydroxyvitamin D3) receptor	VDR	-1.38	-2.52	-1.82	7.98E-04
172	Activity-dependent neuroprotector homeobox	ADNP	-1.97	-3.62	-1.84	1.28E-04
173	Calumenin	CALU	-1.13	-2.08	-1.85	4.17E-03
174	Ubiquitin-conjugating enzyme E2Q family member 1	UBE2Q1	-1.16	-2.15	-1.85	9.56E-03
175	Reticulocalbin 2, EF-hand calcium binding domain	RCN2	-1.07	-1.99	-1.85	1.64E-03
176	ADP-ribosylation factor-like 4C	ARL4C	1.01	-1.84	-1.86	8.49E-03
177	Plakophilin 4	PKP4	-1.01	-1.88	-1.87	6.64E-03
178	Jumonji domain containing 1B	JMJD1B	-1.34	-2.50	-1.87	1.03E-03
179	RAB40B, member RAS oncogene family	RAB40B	-1.36	-2.53	-1.87	5.79E-03
180	F-box protein 11	FBXO11	-1.29	-2.41	-1.87	2.03E-03
181	Protein-kinase, interferon-inducible dsRNA dependent inhibitor	PRKRIR	-1.67	-3.12	-1.87	6.44E-04
182	Proteasome (prosome, macropain) 26S subunit, non-ATPase, 12	PSMD12	-1.09	-2.04	-1.88	2.06E-03
183	Neuropilin (NRP) and tolloid (TLL)-like 2	NETO2	-1.68	-3.16	-1.88	1.35E-03
184	Sorting nexin 4	SNX4	-1.37	-2.58	-1.88	6.28E-03
185	Nucleolar protein 9	NOL9	-1.10	-2.08	-1.89	3.01E-03
186	Solute carrier family 7, member 1	SLC7A1	-1.04	-1.96	-1.89	4.54E-03
187	Nucleoporin 188kDa	NUP188	-1.25	-2.37	-1.89	6.39E-03
188	Myosin phosphatase Rho interacting protein	MPRIP	1.01	-1.89	-1.91	7.14E-03
189	Nuclear receptor subfamily 3, group C, member 1	NR3C1	-1.42	-2.71	-1.91	1.39E-04
190	YTH domain family, member 3	YTHDF3	-1.48	-2.84	-1.91	1.70E-03
191	High-mobility group 20A	HMG20A	-1.19	-2.28	-1.92	7.30E-03
192	Ring finger protein 4	RNF4	1.01	-1.90	-1.92	7.78E-03
193	Caspase 3, apoptosis-related cysteine peptidase	CASP3	-1.02	-1.96	-1.92	6.63E-03
194	Acetyl-Coenzyme A carboxylase alpha	ACACA	1.03	-1.89	-1.93	7.24E-03
195	Chromosome 10 open reading frame 18	C10orf18	-1.42	-2.76	-1.94	3.29E-03
196	ATP-binding cassette, sub-family C (CFTR/MRP), member 4	ABCC4	-1.07	-2.08	-1.94	7.23E-03
197	Myosin phosphatase Rho interacting protein	MPRIP	1.01	-1.92	-1.94	9.28E-03
198	Fibronectin leucine rich transmembrane protein 2	FLRT2	-1.10	-2.14	-1.95	7.98E-04
199	Importin 7	IPO7	-1.08	-2.11	-1.96	4.00E-03
200	Mannosyl glycoprotein beta-1,2-N-acetylglucosaminyltransferase	MGAT2	-1.70	-3.34	-1.97	7.07E-04
201	Ubiquitin specific peptidase 7 (herpes virus-associated)	USP7	-1.42	-2.80	-1.97	2.60E-03
202	Pyridoxal (pyridoxine, vitamin B6) kinase	PDXK	-1.04	-2.06	-1.98	9.33E-03
203	SRY (sex determining region Y)-box 9	SOX9	-1.68	-3.34	-1.99	4.23E-04
204	La ribonucleoprotein domain family, member 1	LARP1	1.06	-1.87	-1.99	9.94E-03
205	Synaptojanin 2	SYNJ2	-1.68	-3.37	-2.00	2.62E-03
206	Chromosome 19 open reading frame 2	C19orf2	-1.41	-2.83	-2.01	1.61E-04
207	Solute carrier family 23 (nucleobase transporters), member 2	SLC23A2	-1.18	-2.38	-2.02	6.34E-03
208	Peptidylprolyl isomerase domain and WD repeat containing 1	PPWD1	-1.32	-2.67	-2.02	5.29E-03
209	mutS homolog 6 (E. coli)	MSH6	-1.31	-2.66	-2.03	1.12E-03
210	Jagged 1 (Alagille syndrome)	JAG1	-1.08	-2.20	-2.03	7.81E-03

211	UDP-Gal:betaGlcNAc beta1,4- galactosyltransferase, polypeptide 5	B4GALT5	-1.21	-2.46	-2.03	6.67E-04
212	Proteasome (prosome, macropain) 26S subunit, non-ATPase, 12	PSMD12	-1.24	-2.52	-2.03	5.71E-04
213	Zinc finger, BED-type containing 5	ZBED5	-1.49	-3.03	-2.04	5.69E-03
214	F-box protein 5	FBXO5	-1.61	-3.27	-2.04	1.60E-03
215	Chromosome 1 open reading frame 116	C1orf116	-1.81	-3.70	-2.04	1.31E-03
216	SH3-domain binding protein 5 (BTK-associated)	SH3BP5	-1.35	-2.78	-2.05	8.23E-03
217	SON DNA binding protein	SON	-1.21	-2.49	-2.06	2.15E-03
218	SEC16 homolog A (S. cerevisiae)	SEC16A	-1.25	-2.58	-2.07	1.99E-03
219	RNA pseudouridylate synthase domain containing 2	RPUSD2	-1.36	-2.81	-2.07	2.54E-03
220	Zinc finger protein 267	ZNF267	-1.27	-2.63	-2.07	4.18E-03
221	Nuclear receptor co-repressor 1	NCOR1	-1.15	-2.40	-2.08	4.66E-03
222	Metadherin	MTDH	-1.05	-2.21	-2.10	5.19E-03
223	SET domain containing 1B	SETD1B	-1.16	-2.44	-2.11	1.64E-03
224	MOB1, Mps One Binder kinase activator-like 3 (yeast)	MOBKL3	-1.20	-2.57	-2.13	1.67E-03
225	Protein tyrosine phosphatase, non-receptor type 11	PTPN11	-1.24	-2.65	-2.14	3.40E-03
226	HIR histone cell cycle regulation defective homolog A	HIRA	-1.23	-2.62	-2.14	4.51E-03
227	SCL/TAL1 interrupting locus	STIL	-1.40	-3.00	-2.14	4.93E-03
228	YTH domain family, member 1	YTHDF1	1.04	-2.07	-2.15	9.76E-03
229	KN motif and ankyrin repeat domains 1	KANK1	-1.51	-3.28	-2.16	2.22E-04
230	Sorting nexin 4	SNX4	-1.23	-2.67	-2.17	3.58E-03
231	DnaJ (Hsp40) homolog, subfamily B, member 4	DNAJB4	-1.34	-2.91	-2.18	3.49E-03
232	RAN binding protein 2	RANBP2	-1.03	-2.27	-2.19	6.46E-03
233	F-box and WD repeat domain containing 2	FBXW2	-1.20	-2.64	-2.20	5.04E-03
234	Coilin	COIL	-2.20	-4.91	-2.24	7.63E-04
235	Myosin X	MYO10	-1.61	-3.62	-2.25	2.30E-06
236	Meningioma (disrupted in balanced translocation) 1	MN1	-1.46	-3.31	-2.26	8.56E-04
237	Nuclear receptor interacting protein 1	NRIP1	-1.62	-3.68	-2.27	3.39E-04
238	Ephrin-B2	EFNB2	-1.19	-2.74	-2.30	5.72E-04
239	SON DNA binding protein	SON	-1.13	-2.60	-2.30	7.93E-04
240	Polo-like kinase 4 (Drosophila)	PLK4	-1.07	-2.47	-2.30	8.52E-03
241	SAC1 suppressor of actin mutations 1-like (yeast)	SACM1L	-1.05	-2.42	-2.32	9.94E-03
242	RIO kinase 2 (yeast)	RIOK2	-2.23	-5.19	-2.32	9.31E-04
243	General transcription factor IIE, polypeptide 1, alpha 56kDa	GTF2E1	-1.56	-3.62	-2.32	1.75E-04
244	Jumonji domain containing 1B	JMJD1B	-1.60	-3.72	-2.32	3.38E-03
245	Core-binding factor, beta subunit	CBFB	-1.63	-3.81	-2.35	6.18E-04
246	Lamin B receptor	LBR	1.05	-2.23	-2.35	9.91E-03
247	MyoD family inhibitor domain containing	MDFIC	-1.19	-2.79	-2.35	6.57E-03
248	Fibronectin type III domain containing 3A	FNDC3A	1.01	-2.33	-2.36	9.25E-03
249	Zinc finger CCCH-type containing 13	ZC3H13	-1.93	-4.58	-2.37	5.00E-03
250	Tryptophan rich basic protein	WRB	-1.76	-4.26	-2.42	9.69E-04
251	Ubiquitin protein ligase E3C	UBE3C	-1.14	-2.79	-2.44	1.60E-03
252	Golgi autoantigen, golgin subfamily a, 3	GOLGA3	-1.14	-2.78	-2.44	2.21E-03
253	Ctr9, Paf1/RNA polymerase II complex component	CTR9	-1.14	-2.82	-2.48	1.40E-03
254	ADP-ribosylation factor guanine nucleotide-exchange factor 2	ARFGEF2	-1.16	-2.88	-2.48	1.59E-03
255	Zinc finger, BED-type containing 4	ZBED4	-1.13	-2.83	-2.51	5.08E-03
256	Hypothetical LOC151162	LOC151162	1.02	-2.46	-2.51	8.58E-03
257	Cullin 3	CUL3	-1.12	-2.84	-2.53	1.85E-03
258	DnaJ (Hsp40) homolog, subfamily B, member 4	DNAJB4	-1.20	-3.04	-2.53	6.85E-03
259	Zinc finger, ZZ-type with EF-hand domain 1	ZZEF1	-1.02	-2.58	-2.54	4.67E-03
260	FERM domain containing 4B	FRMD4B	-1.17	-3.02	-2.58	7.37E-03
261	Ring finger and CHY zinc finger domain containing 1	RCHY1	-1.47	-3.82	-2.59	3.50E-03
262	Chromosome 17 open reading frame 71	C17orf71	-1.94	-5.09	-2.62	7.84E-05
263	Adaptor protein, phosphotyr, PH domain leu zipper containing 1	APPL1	-1.13	-2.97	-2.63	6.16E-03
264	Antigen identified by monoclonal antibody Ki-67	MKI67	1.05	-2.52	-2.64	8.93E-03
265	Transmembrane protein 2	TMEM2	1.07	-2.48	-2.65	7.08E-03
266	Autism susceptibility candidate 2	AUTS2	-1.06	-2.81	-2.67	9.36E-03
267	Rho GTPase activating protein 29	ARHGAP29	-1.04	-2.78	-2.67	3.06E-03
268	Zinc finger, CCHC domain containing 2	ZCCHC2	-1.23	-3.30	-2.68	7.06E-03
269	Squalene epoxidase	SQLE	-1.44	-3.89	-2.70	3.53E-04
270	Ankyrin repeat domain 27 (VPS9 domain)	ANKRD27	-1.13	-3.06	-2.70	8.14E-03
271	RAN binding protein 2	RANBP2	-1.10	-3.04	-2.77	1.60E-03
272	Ring finger protein 219	RNF219	-1.69	-4.75	-2.81	5.28E-04
273	Zinc finger CCCH-type containing 4	ZC3H4	-2.07	-5.85	-2.83	8.07E-05
274	MKL/myocardin-like 2	MKL2	-1.33	-3.79	-2.84	4.30E-03
275	A kinase (PRKA) anchor protein 2	// PALM2 /// PALM[illegible]	1.10	-2.59	-2.86	9.73E-03
276	Eukaryotic translation initiation factor 1A, X-linked	EIF1AX	-1.30	-3.76	-2.89	3.39E-03
277	Leucine zipper protein 1	LUZP1	-1.02	-3.00	-2.93	8.48E-03
278	Embryonic ectoderm development	EED	-1.93	-5.75	-2.99	1.03E-03
279	Purine-rich element binding protein A	PURA	-1.14	-3.41	-2.99	3.68E-03
280	Interferon-induced protein with tetratricopeptide repeats 1	IFIT1	-1.30	-3.92	-3.01	3.86E-04
281	v-crk sarcoma virus CT10 oncogene homolog (avian)	CRK	1.07	-2.83	-3.03	5.54E-03
282	OTU domain containing 4	OTUD4	-1.04	-3.17	-3.06	4.02E-03

283	F-box and leucine-rich repeat protein 5	FBXL5	-1.14	-3.52	-3.09	3.85E-03
284	LEM domain containing 3	LEMD3	1.04	-2.99	-3.12	8.32E-03
285	SS-A/Ro ribonucleoprotein autoantigen 60 kd subunit	TROVE2	-1.00	-3.23	-3.21	8.56E-03
286	REST corepressor 1	RCOR1	1.04	-3.11	-3.22	7.38E-03
287	KIAA0999 protein	KIAA0999	-1.19	-3.87	-3.25	2.95E-03
288	RAN binding protein 6	RANBP6	-1.86	-6.13	-3.30	2.66E-04
289	Chondroitin sulfate synthase 1	CHSY1	-1.22	-4.11	-3.36	2.32E-03
290	Morf4 family associated protein 1-like 1	MRFAP1L1	-1.04	-3.53	-3.41	5.04E-03
291	WD repeat domain 37	WDR37	-1.26	-4.44	-3.51	4.94E-03
292	Cullin 5	CUL5	-1.17	-4.14	-3.54	6.91E-03
293	KIAA0947 protein	KIAA0947	-1.01	-3.75	-3.70	9.34E-03
294	PHD finger protein 20	PHF20	-1.19	-4.44	-3.72	8.79E-03
295	Hbc647 mRNA sequence	SLC30A1	1.00	-3.78	-3.78	8.59E-03
296	SNF related kinase	SNRK	1.06	-3.65	-3.88	9.61E-03
297	Dedicator of cytokinesis 4	DOCK4	-1.20	-4.73	-3.93	9.72E-03
298	Jumonji, AT rich interactive domain 2	JARID2	-1.18	-4.68	-3.95	7.58E-03
299	KIAA0232	KIAA0232	-1.15	-4.62	-4.01	4.47E-03
300	G protein-coupled receptor 125	GPR125	1.08	-3.77	-4.08	9.66E-03
301	Nucleoporin 50kDa	NUP50	-1.26	-5.36	-4.27	5.36E-04
302	CREB binding protein	CREBBP	1.07	-4.12	-4.40	6.66E-03
303	Wings apart-like homolog (Drosophila)	WAPAL	-1.42	-6.24	-4.40	2.42E-03
304	Phosphoinositide-3-kinase, regulatory subunit 4	PIK3R4	-1.03	-4.59	-4.47	2.79E-03
305	CLP1, cleavage and polyadenylation factor I subunit, homolog	CLP1	1.02	-5.07	-5.19	4.15E-03
306	Ras responsive element binding protein 1	RREB1	-1.06	-6.17	-5.84	4.17E-03
307	Fem-1 homolog b (C. elegans)	FEM1B	1.10	-5.91	-6.50	8.06E-03
308	Asp (abnormal spindle) homolog, microcephaly associated	ASPM	1.12	-7.67	-8.55	6.90E-03
